# Effects of an Ad Libitum Consumed Low-Fat Plant-Based Diet Supplemented with Plant-Based Meal Replacements on Body Composition Indices

**DOI:** 10.1155/2017/9626390

**Published:** 2017-03-28

**Authors:** Boštjan Jakše, Stanislav Pinter, Barbara Jakše, Maja Bučar Pajek, Jernej Pajek

**Affiliations:** ^1^Barbara Jakše s.p., SI-1230 Domžale, Slovenia; ^2^Faculty of Sport, University of Ljubljana, Gortanova 22, SI-1000 Ljubljana, Slovenia; ^3^University Medical Center Ljubljana, Zaloška 2, SI-1525 Ljubljana, Slovenia

## Abstract

*Objective*. To document the effect of a diet free from animal-sourced nutrients on body composition indices.* Methods*. This was a nonrandomized interventional (*n* = 241)-control (*n* = 84) trial with a 10-week, low-fat, plant-based diet supplemented with two daily meal replacements. The meals were allowed to be eaten to full satiety without prespecified calorie restrictions. Control subjects received weekly lectures on the rationale and expected benefits of plant-based nutrition. Body composition indices were measured with bioimpedance analysis.* Results*. Relative to controls, in cases, postintervention body fat percentage was reduced by 4.3 (95% CI 4.1–4.6)% points (a relative decrement of −13.4%), visceral fat by 1.6 (95% CI 1.5–1.7) fat cross-sectional surface units, and weight by 5.6 kg (95% CI 5.2–6), while muscle mass was reduced by 0.3 kg (95% CI 0.06–0.5) with a relative increase of muscle mass percentage of 4.2 (3.9–4.4)% points. Analysis of covariance showed significantly larger adjusted fat reductions in cases compared to controls. Late follow-up revealed further weight loss in 60% of cases and no significant change in controls.* Conclusions*. Low-fat, plant-based diet in free-living nonresidential conditions eaten ad libitum enables significant and meaningful body fat reductions with relative preservation of muscle mass. This trial is registered with NCT02906072, ClinicalTrials.gov.

## 1. Introduction

Weight gain and adiposity are the two single most important causes of metabolic syndrome, type 2 diabetes mellitus, arterial hypertension, osteoarthritis, and certain cancers and represent one of the main global public health problems [1]. The scale of the problem is represented by the fact that age-standardized global prevalence of obesity increased approximately threefold in the last four decades and it may reach 18–21% by 2025 [2]. Besides a sedentary life-style, diets with suboptimal composition and caloric excess are the main contributory factors to this unprecedented rise of obesity [3]. Experience from work in weight management programs however shows that most people do not link the problems of obesity and adiposity to diet composition and a possible dependence on the western-style diet but may attribute these issues to personal factors like lack of will-power, overeating, and financial constraints.

Recently, popular diets low in carbohydrates (LC) and very low carbohydrate- (VLC-) ketogenic diets, high in animal-based protein, were shown to result in weight reduction and modification of some cardiovascular risk factors [4]. However, reports from some studies using animal models and humans have suggested detrimental health effects such as accelerated atherosclerosis, poorer endothelial function and elevated arterial stiffness, elevation of some cardiovascular risk factors such as fibrinogen, lipoprotein (a), and C-reactive protein (CRP), and association with elevated all-cause mortality risk [5–8]. A suggested alternative dietary paradigm is to avoid animal derived nutrients and to predominantly use plant-based dietary sources. Studies involving a low-fat plant-based dietary intervention have shown reductions in body weight, normalization of body mass index (BMI), and improvements in serum lipid profile and insulin sensitivity with possible reductions in cardiovascular morbidity [9–12].

An additional strategy for diets optimized towards weight control and optimal long-term health may involve the use of meal replacements. Previous studies with caloric restriction have shown that, by supplementing 2 out of 3 conventional meals with a meal replacement, a larger weight reduction and a better compliance with diet plan could be achieved compared to isocaloric conventional diets [13, 14]. Long-term compliance, adherence to diet plans, and preservation of weight reduction are a significant concern with weight-loss interventions. The rate of failure of various weight-reducing interventions and the probability of not attaining target weight in obese people of both genders may reach up to 99.8% [15, 16]. Meal replacements are formulated to mimic a low-caloric conventional meal. They may be easily and quickly prepared in the form of shakes or smoothies and enable easier meal planning through the day in everyday life. Inclusion of meal replacements may therefore represent one of the solutions for improving the long-term success of weight-reducing diets in free-living subjects [17, 18]. Furthermore, the inclusion of meal replacement supplementation in an outpatient weight-reducing program was proven safe as regards to liver, kidney, and bone mineral density markers even at higher protein group (2.2 g protein/kg of lean body mass) in isocaloric weight-loss meal plan over the 12-month period [19].

In the present study we report the effects of an ad libitum consumed plant-based diet involving 3 meals combined with 2 additional (plant-based) meal replacements on the body composition of free-living participants. We hypothesized that the application of an ad libitum low-fat plant-based diet without a prespecified calorie restriction would induce clinically relevant weight loss, predominantly via reduction of fat mass with minimal changes of fat-free mass. The impact of low-fat plant-based diets supplemented with meal replacements on body composition was so far understudied and our report reveals novel insights into the potential of this dietary approach to enable body fat reduction and muscle mass preservation.

## 2. Materials and Methods

### 2.1. Study Design

This study was designed as an open-label, nonrandomized, controlled interventional trial, followed by a postintervention survey in a nonresident diet-optimizing program. The primary outcome of the study was a change in body fat mass, which is in agreement with recent considerations of measuring outcomes in weight intervention studies [20]. Secondary outcomes included changes in body weight, muscle mass, and visceral fat mass. We included participants older than 18 years who took part in the program in the period from January 2011 through January 2016 and who completed a 10-week diet-optimizing program as detailed below. Pregnant women, patients with dietary restrictions from a treating physician, and patients with active malignant disease and professional athletes were excluded from the study. Participants who, after an introductory program presentation, opted not to follow the proposed dietary intervention including meal replacements, but only to attend the lectures on health effects of a low-fat plant-based diet, adjusted their diet by their own judgment, and attended weekly body composition follow-up measurements, served as controls. Subjects were invited to participate after spontaneously attending the introductory lecture on the basis of information gathered by personal informal contacts and referrals from previous program attendees and by information and posted results given on the Internet social networks. Each participant signed an informed consent statement for inclusion in the study. The study was approved by Slovenian Medical Ethics Committee (approval document number 0120-265/2016-3).

### 2.2. Study Protocol

Baseline evaluation included a questionnaire-based survey of dietary habits, physical activity, comorbid diseases, and personal goals associated with participation in the diet-optimizing program. A questionnaire developed by the investigators was used whereby dietary habits were assessed with items defining current intake of main food groups, a typical daily meal plan, dietary supplements, and diet related health issues. Physical activity was assessed with three items in the questionnaire defining the number of exercise units per week, the type of exercise, and the time spent. Body composition was assessed at baseline, once weekly at regular follow-up meetings, and at the end of the 10-week program by an 8-electrode bioimpedance body composition monitor (Tanita BC-601F; Tanita Corporation, Tokyo, Japan). Body composition assessment measurements included body weight, BMI, body fat mass percentage relative to total body mass, visceral fat area (in arbitrary units associated with abdominal visceral fat cross-sectional area (each unit equates to 10 cm^2^ of visceral fat)), muscle mass, total body water, mineral bone mass, and estimated basal metabolic rate.

The initial questionnaire survey revealed that at baseline participants ingested on average 2–4 meals reflective of a typical Western-type diet: most meals were composed of animal-source nutrients (cow-milk, yoghurt, cheese, cottage-cheese, meat from various sources, eggs, and fish) and refined wheat flour based nutrients (bread, pasta, and pastry). Food preparation included various vegetable oils and fats. Unrefined and whole plant nutrients were largely absent from most meals and a minority of meals included portions of fruit and vegetables. These data indicate that our participants were in general without any significant personal preference towards plant-based diets before study entry. No ideological or philosophical arguments towards vegetarian diet choices or against animal use were mentioned.

The dietary intervention was executed in free-living conditions with participants engaging in their regular daily work and social activities. The plant-based dietary plan included 3 conventional meals based on starch nutrients (potatoes, sweet potatoes, rice, oatmeal, whole-grain pasta, beans, peas, lentils, and similar ones), fruits (seasonal fruits and various berries), and nonstarch vegetables (color and leafy vegetables). Spices and tomato sauce (without oil) and one regular-sized spoon of flaxseed were recommended as well. The participants were recommended to consume no more than 5-6 grams of salt per day. All milk and dairy products, vegetable oils, and fats were excluded from the diet. Meat was allowed (but not recommended) once weekly to relieve social pressures on participants which they often encountered from their circle of influence (i.e., family, friends, and coworkers) when changing the diet to plant-based sources. The total macronutrient composition of the intervention diet was approximated to 15% protein, 70% carbohydrates, and 15% fat. Dietary fiber content was approximated to 40–45 g per day, which is in alignment with research on plant-based dieters [21]. Both meal replacements and conventional meals were allowed to be consumed ad libitum (to full satiety). No calorie count or limits were instituted. The exact composition of the intervention diet is given in Table 1.

All participants were followed at weekly intervals. Evaluation of dietary diaries describing meal composition and food intake self-reports in the form of meal photographs were used to monitor compliance and correct and adjust deviations from the dietary plan and to help participants prepare the meals according to the dietary plan. Weekly lectures about the rationale and guidance on attaining the low-fat plant-based diet were given to all subjects (intervention and control group). Participants were encouraged to engage in at least two weekly sessions of 45 minutes of moderate-intensity exercise activity. Guided 45-minute moderate-intensity exercise sessions were organized for those who opted in and written exercise program to mimic guided sessions at moderate intensity was given to participant not attending the guided sessions. During the 10-week program 80% of all participants (intervention and control group) attended these exercise sessions.

### 2.3. Statistical Analysis

Results are presented as means ± SD for normally distributed and as medians (range) for nonnormally distributed variables. Differences between groups were tested with *t*-test for unpaired and paired samples, as appropriate. Mann–Whitney and Wilcoxon Signed Rank tests were used for nonnormally distributed data. Chi-square test was used for categorical variables. Differences between cases and controls were tested with analysis of covariance (general linear model), with adjustment for baseline variable status, age and sex. IBM SPSS statistics application was used for all analyses; *p* < 0.05 was taken as the limit of statistical significance.

## 3. Results and Discussion

### 3.1. Results

**Figure 1:**
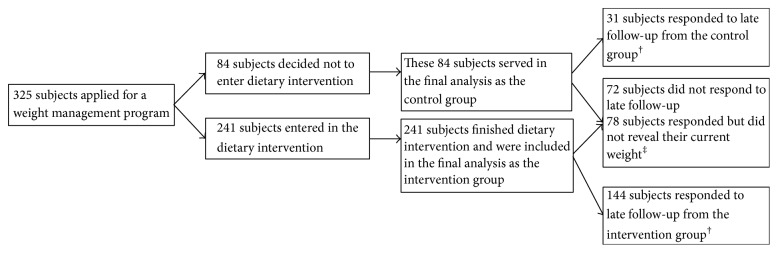
Participant flow through study phases. ^†^Late follow-up was done at the median period of 17.4 months after finish of the dietary intervention; 31 subjects from control group and 144 subjects from intervention group responded and revealed their current body weight; ^‡^150 participants from both groups did not respond or did not want to reveal their current weight.

325 subjects were included in the analysis, 282 (87%) females and 43 (13%) males with average age of 41.2 ± 12 years. The flow of participants through the study phases is shown in Figure 1. The demographic variables of the control (*N* = 84, 26%) and the intervention group (*N* = 241, 74%) are shown in Table 2. There were no drop-outs from any of the groups. Baseline body composition indices are given in Table 3. Results are given separately for women and men.

Differences in body composition between final (study end) and baseline values are given in Table 4 for control and intervention groups, respectively. Since the intervention group and the control group were not balanced in weight and body fat at baseline, the effect of the intervention was finally tested with analysis of covariance, with adjustment for baseline variable value, age and sex.  Table 5 shows the B coefficients which represent the adjusted difference between intervention and control group.

The intervention group lost a significantly larger amount of weight, body fat, and visceral fat. In addition, the relative proportion of muscle mass and total body water increased relative to the control group. Even though the intervention group lost a significant amount of body weight and fat, muscle mass loss was negligible and this contributed to an increase of the relative proportion of muscle mass.

**Figure 2:**
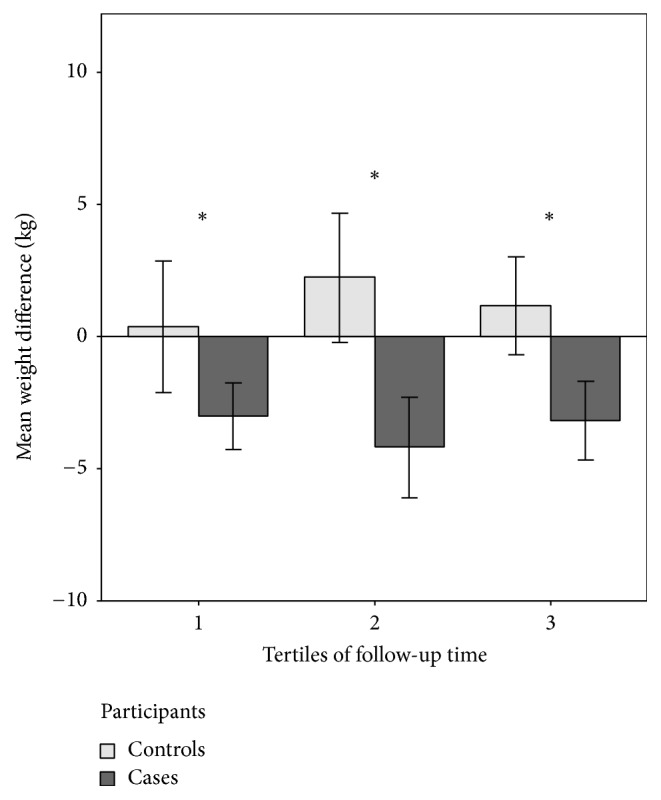
Weight change of cases and controls at the late follow-up. First tertile of follow-up was 0–13 months after the end of the program and second tertile 13–25 months. ^*∗*^*p* < 0.05 for the difference between cases and controls in the same tertile. Error bars denote 95% confidence intervals.

In the period of 10th–20th May 2016, at the median time lag of 17.4 months (range 3.4–64.2) after the end of the program, subjects were surveyed to obtain data on current body weight. Seventy-two participants (22%) did not respond, 78 participants (24%) responded but did not want to reveal their current weight, and 175 participants (54%) responded (31 participants from the control group and 144 participants from the intervention group). Mean body weight change from the end-of-program to time of follow-up survey was 1.1 ± 3.6 kg (*p* = 0.09) and −3.5 ± 5.5 kg (*p* < 0.001) for participants from the control group and participants from the intervention group, respectively. Mean changes of follow-up weight according to the time of follow-up are displayed in Figure 2.

### 3.2. Discussion

In the present study we report the effects of a dietary intervention combining two potentially efficient approaches for optimization of human diets: a low-fat, plant-based diet and plant-based meal replacements. Our key findings include a significant reduction in fat mass, visceral fat, and body weight and, importantly, a negligible decrement in absolute muscle mass with an increment in muscle mass proportion. Improvements were statistically significantly different in the intervention group compared to the control group; however, even in the control group some significant beneficial effects such as minor reductions in body weight and fat mass were observed.

Studies with vegetarian diet interventions were shown to result in a mean change in body weight of −3.4 kg (95% CI −4.4 to −2.4 kg) [22]. When compared to these meta-analysis data with studies of various duration, our study intervention was able to provide a mean weight reduction above this range: the intervention group lost an average of 5.6 kg, with an average reduction of 7.3 kg in the subsample of obese subjects. Since the reduction in muscle mass was minimal (−0.3 kg on average and −0.9 kg in the obese subsample) we can infer that the majority of the weight reduction was accounted for by a reduction of fat mass. Fat mass proportion was reduced by 4.3% absolute percent points and a significant reduction in visceral fat was also demonstrated. The reduction of visceral fat is especially important since there is evidence that abdominal obesity is associated with elevated cardiovascular risk and mortality [23, 24]. Low-fat plant-based dietary interventions, even without preplanned caloric restriction, such as in our study, usually result in the reduction of caloric intake in the range of 11–31%, mostly due to a lower caloric density (increased fiber and reduced fat intake) [25, 26]. A previous study in a large resident dietary program with a low-fat starch-based plant diet showed efficient weight loss reaching an average reduction of 1.4 kg in a 10-day program [27]. The body weight-reducing potential of a low-fat plant-based (high carbohydrate) diet in overweight individuals was further confirmed in a corporate setting [10] and in an overweight population based sample [28]. There is however a lack of data concerning the composition of the body weight lost (fat mass or fat-free mass) induced via plant-based diets.

In this study we documented that muscle mass was well preserved, despite a significant and relatively large weight reduction and a relatively moderate protein content (approximately 15% of total daily calories from protein). Muscle mass loss in the intervention group constituted only 3.6% of total weight loss and 12.3% of weight loss in the subsample of obese subjects. Preservation of muscle mass in weight reduction programs is important due to the association of lean and muscle mass with resting metabolism level [29, 30]. Given that fat-free mass represents a key determinant of the magnitude of resting metabolic rate, it follows that a decrease in lean muscle tissue could hinder the progress and maintenance of weight loss. Thus, the loss of fat mass while maintaining muscle mass and resting metabolic rate seems desirable [31]. Reductions of fat-free mass were significant in some of the weight reduction studies and it seems that loss of muscle mass is mainly associated with the severity of energy restriction [29, 32, 33]. We believe that in our study two main factors were preventing the loss of muscle mass: the ad libitum food intake, which prevented severe caloric restriction, and the promotion of exercise, which may limit the proportion of muscle mass lost during a weight reduction program [34–36].

Meal replacement usage in weight management programs is associated with simplification of diet preparation and may improve the long-term success of a diet plan [17, 37, 38]. Our cohort has managed to keep high short-term compliance since there were no drop-outs from the intervention program. Long-term follow-up after the median period of 17.4 months showed that 60% of the intervention group subjects who responded to the query and were willing to provide data on their current weight on average lost an additional 3.3 kg of body weight. This, however, cannot be assumed for the 40% of the subjects from the intervention group who did not respond, so, at best, we can claim a moderate long-term success of the program.

Our study has some obvious limitations inherent to studies of free-living subjects. The adherence to a diet plan could only be assessed from the subjective diaries and food intake self-reports, which are prone to underreporting of energy intake, especially in obese people [39, 40]. Therefore we may consider this study primarily to offer evidence on the effectiveness of a low-fat plant-based diet prescription. Additionally, our intervention was not limited to diet only, since participants were encouraged to increase their physical activity levels during the lectures and moderate-intensity 45-minute exercise sessions were offered to participants once to twice weekly. Although in general an increase in physical activity alone may have only a small impact on obesity [41], we cannot fully dissociate the effects of the dietary intervention from the possible additional effects of enhanced physical activity in our study. The food diaries of all participants were reviewed on a weekly basis and this may have been an additional stimulus for better success of the program, since behavioral measures such as food diaries may account for a significant part of the success observed with weight management programs [42]. Finally, body composition was assessed with the usage of bioimpedance technology and although 8-electrode bioimpedance is recognized as a valid method to evaluate body composition changes in adult weight-loss studies [20, 43], usage of dual energy X-ray absorptiometry, which was extensively validated against underwater weighing, is used more often [20, 33]. However, due to logistic demand of weekly follow-up at various locations, the usage of bioimpedance was the single best option for our study design.

## 4. Conclusions

Our study documents the body composition effects of the combination of two dietary approaches for human weight loss—an ad libitum consumed low-fat plant-based diet, which is supplemented with meal replacements. Prescription of this dietary intervention to free-living subjects for a duration of 10 weeks and without prespecified calorie counts and calorie restrictions resulted in significant reductions in body fat, visceral fat, and total body weight. The magnitude of weight reduction was above the average weight reduction reported for vegetarian diet interventions [22]. Muscle mass of participants was reduced minimally. The postintervention follow-up showed an additional weight reduction in the participants willing to report their body mass, suggesting moderate success of the program in the long-term. We conclude that our intervention showed significant and clinically relevant positive effects on body composition. Future studies are needed to explore the impact of low-fat plant-based diets on cardiovascular risk markers, clinical morbidity, and mortality end-points.

## Figures and Tables

**Table 1:** Composition of the intervention diet.

Meal	Dietary plan	Macronutrient composition^†^	Calorie intake^‡^
Breakfast	Meal replacement^*∗*^ (serving size 2 scoops in water or soy milk), ground flaxseed (1 table spoon), and oat meal (ad libitum)	15% protein, 60% carbohydrate, 25% fat	250 (200–300) kcal
Morning snack	3 dcl of smoothie (spinach, berries, or other seasonal local fruits) or 2-3 portions of seasonal fruits	10% protein, 80% carbohydrates, 10% fat	150 (100–200) kcal
Lunch	Centered around starches; 4-5 food groups (whole grains: brown rice, pasta, buckwheat, millet, and corn; legumes: lentil, bean, and pea; tubers and pumpkins: potato and sweet potato; brassica: broccoli, cauliflower, kale, and cabbage; color and leafy vegetables: tomato, green salad)	15% protein, 80% carbohydrate, 5% fat	500 (450–550) kcal
Afternoon snack	Sandwich (whole-grain bread, humus, tomato, kale, or cabbage) or millet with mixed berries or seasonal local fruits (if not already for morning snack)	8–20% protein, 68–88% carbohydrate, 8–12% fat	250 (200–300) kcal
Dinner	Mixed green salad: green leafy vegetables, boiled potato, tomato, walnuts, or what was left from lunch and always meal replacement	17% protein, 60% carbohydrate, 23% fat	300 (200–400) kcal

^†^Overall (on average) estimated macronutrient composition: 15% protein (54 g), 70% carbohydrate (272 g), 15% fat (24 g), and 1450 (1250–1750) kcal.

^‡^Calorie estimation was based on recommended dietary meal plan and dietary diaries and photographs, using ESHA Food Processor Nutrition Analysis Software (http://www.esha.com). ^*∗*^Meal replacement used was Herbalife European Free From Vanilla® nutritional powder.

**Table 2:** Demographic characteristics of control and interventional groups.

Parameter	Whole sample	Control group	Intervention group	*p*
	(*N* = 325)	(*N* = 84)	(*N* = 241)	
Age (years)	40 (18–71)	41 (18–71)	40 (19–69)	0.98
Sex (female/male (%))	282/43 (87%/13%)	74/10 (88%/12%)	208/33 (86%/14%)	0.68
Height (cm)	167 (152–200)	168 (153–200)	167 (152–193)	0.9
Weight (kg)	77.7 (48.8–149.1)	73.7 (49.2–139.3)	79.3 (48.8–149.1)	0.02
Smoking (*n* (%))	19 (6%)	4 (5%)	15 (6%)	0.79
Married or living with a partner (*n* (%))	250 (77%)	57 (68%)	193 (80%)	0.02
University educational level (*n* (%))	117 (36%)	21 (25%)	96 (40%)	0.02
Frequent exercisers^†^ (*n* (%))	26 (8%)	3 (4%)	23 (10%)	0.08

For normally distributed variables the data are given as mean ± SD and for nonnormally distributed variables as median (range). ^†^Habitual personal workout more than 3 times per week.

**Table 3:** Baseline body composition indices.

Parameter	Females		Males	
	Control group	Intervention group	Control group	Intervention group
	(*N* = 74)	(*N* = 208)	(*N* = 10)	(*N* = 33)
Weight (kg)	72.8 (49.2–118.1)	76.6 (48.8–149.1)^*∗*^	83.7 (63.6–139.3)	91 (65.5–140)
BMI^†^	26.4 (17.7–42.6)	27.5 (18.6–47.7)	23.2 (20.1–34.8)	28.9 (21.1–46.2)
Body fat (%)	34.3 (16.1–46.7)	37.1 (18.1–53.8)^*∗∗*^	15.5 (6.3–28.3)	24.4 (11.3–37.4)^††^
Visceral fat (arbitrary units)	5.5 (1–13)	7 (1–16)^*∗*^	4.5 (1–16)	9 (1–24)
Total body water (l)	48.5 (39.9–61.3)	46.9 (34.6–60.3)^*∗∗*^	60.1 (49.6–66.8)	53.4 (46.4–64.7)^††^
Muscle mass (kg)	45.5 (35.5–65.1)	46.2 (34.4-79.4)	71.2 (52–95.9)	65.8 (55.2–83.4)
Muscle mass percent (%)^‡^	62 (51–79)	60 (44–78)^*∗∗*^	80 (68–89)	72 (60–84)^††^
Estimated basal metabolic rate (kCal)	2310 (1750–3270)	2330 (1770–4510)	3390 (1910–4930)	3250 (2790–4270)

Since most variables were non-normally distributed the data are presented as median (range). ^†^BMI, body mass index. ^††^*p* = 0.06 for the difference between the control and intervention group in the male sex category. ^‡^Muscle mass percent, percent of muscle mass relative to body weight. ^*∗*^*p* < 0.05 for the difference between the control and interventional group in the same sex category. ^*∗∗*^*p* < 0.01 for the difference between the control and interventional group in the same sex category.

**Table 4:** The differences in body composition indices between final and baseline study values.

Parameter	Control group	Interventional group	*p* for difference between groups
	(*N* = 84)	(*N* = 241)	
*Whole sample regardless of BMI*			
Weight change (kg)	−1.2 (−1.6 to −0.8)	−5.6 (−6 to −5.2)	<0.001
Body fat% change (difference in absolute % points)	−0.4 (−0.7 to −0.2)	−4.3 (−4.6 to −4.1)	<0.001
Relative body fat change (%)	−0.9 (−2.2 to 0.3)	−13.4 (−14.3 to −12.5)	<0.001
Visceral fat change (arbitrary units)	−0.1 (−0.2 to 0.01)	−1.6 (−1.7 to −1.5)	<0.001
Muscle mass change (kg)	−0.4 (−0.7 to −0.1)	−0.3 (−0.5 to −0.06)	0.25
Muscle mass% change (% points)^†^	0.4 (0.1 to 0.7)	4.2 (3.9 to 4.4)	<0.001
Total body water change (l)	0.3 (0.1 to 0.5)	3.1 (2.9 to 3.3)	<0.001

*BMI 30 or more*			
Weight change (kg)	−1.9 (−2.7 to −1.1)	−7.3 (−8 to −6.6)	<0.001
Body fat% change (difference in absolute % points)	−0.5 (−1 to −0.05)	−3.8 (−4.2 to −3.4)	<0.001
Relative body fat change (%)	−1.1 (−2.2 to 0.03)	−9.6 (−10.8 to −8.5)	<0.001
Visceral fat change (arbitrary units)	−0.4 (−0.6 to −0.2)	−2 (−2.2 to −1.8)	<0.001
Muscle mass change (kg)	−0.7 (−1.4 to 0.05)	−0.9 (−1.3 to −0.5)	0.85
Muscle mass% change (% points)^†^	0.004 (−0.002 to 0.01)	0.04 (0.03 to 0.04)	<0.001
Total body water change (l)	0.2 (−0.2 to 0.6)	2.8 (2.5 to 3.1)	<0.001

Mean differences with 95% confidence intervals are shown. In the BMI 30 or more there were 30 subjects in the control group and 89 subjects in interventional group. ^†^Muscle mass change is given in % points relative to total body mass.

**Table 5:** Analysis of covariance with adjustment for baseline values, age, and sex.

Parameter	Adjusted difference between interventional and control groups	95% confidence interval	*p*
Final weight (kg)	−4.5	−2.8 to −6.2	<0.001
Final body fat% (% points)	−4.9	−3.7 to −6.1	<0.001
Final visceral fat (arbitrary units)	−2	−1.5 to −2.5	<0.001
Final muscle mass (kg)	0.5	−0.6 to 1.5	0.37
Final muscle mass percent (% points)	4.6	3.4 to 5.8	<0.001
Final total body water (l)	3.6	2.6 to 4.6	<0.001

For every dependent final variable in the first column the difference between interventional and control group was adjusted for baseline value of that variable, age and sex (analysis of covariance with general linear model ANOVA). Results and statistical significance remained materially unchanged when analyses were repeated separately for both genders.
